# Lost in fragmentation — care coordination when somatic symptoms persist: a qualitative study of patients’ experiences

**DOI:** 10.3399/BJGP.2021.0566

**Published:** 2022-09-21

**Authors:** Hieke Barends, Femke Botman, Ella Walstock, Nikki Claassen-van Dessel, Johannes C van der Wouden, Tim olde Hartman, Joost Dekker, Henriëtte E van der Horst

**Affiliations:** Department of General Practice, Amsterdam UMC, Vrije Universiteit Amsterdam and Amsterdam Public Health Research Institute, Amsterdam.; Department of General Practice, Amsterdam UMC, Vrije Universiteit Amsterdam and Amsterdam Public Health Research Institute, Amsterdam.; Department of General Practice, Amsterdam UMC, Vrije Universiteit Amsterdam and Amsterdam Public Health Research Institute, Amsterdam.; Department of General Practice, Amsterdam UMC, Vrije Universiteit Amsterdam and Amsterdam Public Health Research Institute, Amsterdam.; Department of General Practice, Amsterdam UMC, Vrije Universiteit Amsterdam and Amsterdam Public Health Research Institute, Amsterdam.; Department of Primary and Community Care, Donders Institute for Brain, Cognition and Behaviour, Radboud University Nijmegen Medical Center, Nijmegen.; Amsterdam Public Health Research Institute, Amsterdam, and Department of Rehabilitation Medicine and Department of Psychiatry, Amsterdam UMC, Vrije Universiteit Amsterdam, Amsterdam.; Department of General Practice, Amsterdam UMC, Vrije Universiteit Amsterdam and Amsterdam Public Health Research Institute, Amsterdam.

**Keywords:** coordination of care, general practice, medically unexplained symptoms, persistent somatic symptoms, primary care, qualitative research

## Abstract

**Background:**

GPs can play a central role in the care of patients with persistent somatic symptoms (PSS). To date, little is known about these patients’ experiences relating to their coordination of care.

**Aim:**

To explore the experiences of patients with PSS relating to coordination of care — in particular by their GP — during their illness trajectory.

**Design and setting:**

This qualitative study was carried out from January to April 2019 in the Netherlands as part of a multicentre prospective cohort study on the course of PSS (PROSPECTS).

**Method:**

Thematic content analysis of 15 interviews.

**Results:**

Three themes were identified: care fragmentation during the diagnostic trajectory; transition from the search for a cure to coping; and reframing to coping: GPs’ role in facilitating supportive care. Patients experienced a lack of collaboration from healthcare workers during the diagnostic trajectory. Guidance by their GP in a process of shared decision making was positively valued by patients. Moving the focus from searching for a cure to coping with symptoms was described as a ‘personal endeavour’, made even more challenging by the ongoing uncertainty experienced by patients. When reframing to coping, the extent to which patients felt aligned with their GP played an important role in whether their supportive care request was met.

**Conclusion:**

Patients experienced difficulties when navigating the diagnostic trajectory and shifting to coping. The findings of this study underline the importance of collaboration between GPs and other healthcare professionals during the diagnostic trajectory. The authors recommend that GPs provide proactive guidance and are sensitive to patients who shift to coping by providing them with supportive care in a process of shared decision making.

## INTRODUCTION

GPs regularly encounter patients who present with somatic symptoms, the origin of which remains unclear after adequate history taking, physical examination, and, if warranted, additional investigations.^1^^–^3 In primary care, these symptoms are commonly referred to as ‘medically unexplained symptoms’. A recently introduced and more appropriate and patient-centred term — placing less emphasis on the mind–body dualism in the origin of symptoms — is persistent somatic symptoms (PSS).^4^^–^6 In the current study, the term PSS is used throughout. Patients with PSS account for a substantial proportion of frequent attenders in primary care.^7^ Having PSS is associated with elevated psychological distress, functional impairment,^8^^,^^9^ and high medical care utilisation,^10^^,^^11^ putting patients at risk of iatrogenic harm.^12^^–^15

As a diagnosis is an expected outcome of a medical interaction, both doctors and patients can feel frustrated and lost without one. A cultural result of modern medicine is that there is a compelling expected narrative in the management of illness. Arthur Frank outlined this as the ‘restitution narrative’: most individuals make sense of their illness by approaching it as a narrative. In the ‘restitution narrative’ every disease has a name — a diagnosis — and is preferably followed by a successful cure resulting in a happy ending.^16^ Patients need to make sense of their illness stories in a culture that prefers restitution stories. In the case of PSS, a diagnosis and cure often remain absent. This can cause deep discomfort and may hamper the provision of adequate long-term care for individuals with PSS.

GPs encounter challenges in the management of care for patients with PSS. They tend to find these symptoms difficult to manage,^17^ experience less satisfaction,^18^^,^^19^ and have a high workload in caring for patients with PSS.^20^ The biomedical disease model, in which the ultimate aim is to correct disease and restore normal functioning (which is in line with the culturally appropriate ‘restitution narrative’), still prevails in medical education and practice.

Most GPs struggle with the incongruence between the dominant disease model and the reality of patients with PSS.^21^ Despite GPs’ frequent struggle with PSS management, most GPs consider it their role to manage the care of these patients in primary care services.^17^ In current guidelines, GPs play a central role in the guidance and management of patients with PSS in healthcare systems with a gatekeeper system such as the Netherlands.^22^^–^25

Many studies on the experiences of patients with PSS focus on the interpersonal communication between the GP and the patient, and what patients expect from their GPs. These studies highlight that patients regularly feel dissatisfied with the care they receive, frequently experiencing prejudices^8^^,^^26^ or being told by their doctor that ‘there is nothing wrong’ — which does not match their experience of having severe symptoms that affect their daily lives.^27^ They long for an explanation for their symptoms^28^ and would like to be more involved in agenda setting and treatment decisions — focusing on what matters to them.^8^^,^^26^^,^^29^

**Table:** How this fits in

In healthcare systems where the GP acts as a gatekeeper, such as the Dutch healthcare system, GPs can play a central role in providing care for patients with persistent somatic symptoms (PSS). To optimise coordination of care and identify best practices it is necessary to understand how patients with PSS currently experience coordination of care. In this study patients frequently reported that they experienced fragmented care during the diagnostic trajectory and mentioned challenges in finding support to cope with symptoms when they made the transition from the search for a cure to coping. The findings of this study underline the importance of collaboration between GPs and other healthcare professionals when providing care for patients with PSS. The authors of this study recommend that GPs provide proactive guidance during the diagnostic trajectory and are sensitive to patients who shift to coping by providing them with supportive care in a process of shared decision making.

Ensuring optimal care coordination in patients with PSS may be challenging for both patients and GPs, especially when many care providers are involved. With the term care coordination, the authors of this study mean:

*‘… the deliberate organization of patient care activities between two or more participants (including the patient) involved in a patient’s care to facilitate the appropriate delivery of health care services’.*^32^

When referred to specialist care for further examination, patients with PSS may, for example, end up in a *‘collusion of anonymity’*. This phenomenon, first described by Michael Balint in the 1950s, refers to a situation in which *‘the patient is passed from one specialist to another with nobody taking responsibility for the whole person’*.^33^

In the interviews that the authors of the current study conducted with patients with PSS, the patients reflected on their illness trajectories. Fragmented care during these trajectories and the search for supportive care played an important role in their illness narratives.

Although the initial aim and purpose of these interviews was to explore patients’ experiences with fluctuations in their PSS over the course of their illness trajectories,^34^^,^^35^ it was decided as a secondary aim to explore patients’ experiences of care coordination in more detail. To the best of the authors’ knowledge, no prior study has focused primarily on how patients with PSS experience coordination of care — in particular by their GPs — during their illness trajectory.

## METHOD

This qualitative study is part of a larger prospective cohort study that monitors the course of symptoms and functional health of patients with PSS (Box 1).^30^^,^^31^ Semi-structured (in-depth) interviews were conducted to obtain information about the experiences of patients with PSS over the course of their illness trajectory.

**Box 1.** The PROSPECTS study

The PROSPECTS study is a Dutch longitudinal cohort study following patients (*n* = 325) with persistent somatic symptoms (PSS). Patients with PSS aged between 18 and 70 years were recruited from general practices (*n* = 218) and specialised PSS programmes in secondary and tertiary care organisations (*n* = 107) across the Netherlands in 2013–2015. Initially patients were followed over a period of 3 years with five measurements in time (baseline, 6-, 12-, 24- and 36-months’ follow-up). In 2017, the follow-up period was extended and the study is still ongoing. Baseline characteristics and information on the recruitment process and first 2 years of follow-up have been published elsewhere.^30^^,^^31^ Definition of PSS: PSS was defined as the presence of physical symptoms that had lasted at least several weeks and for which no sufficient explanation was found after proper medical examination by a physician. This is in line with the current Dutch multidisciplinary and general practice guidelines for medically unexplained physical symptoms.^23^^,^^25^

### Participants

For the interviews, those patients from the PROSPECTS study who completed the 3-year follow-up and gave informed consent to be contacted for future research were invited to take part. Purposive sampling was used to ensure a diversity of participants in terms of the nature of their symptoms, age, sex, social characteristics (educational level, location), and recruitment setting (general practice, specialised PSS programme). Because of the main aim of the interviews,^35^ patients with either clinically relevant fluctuations or clinical stability (based on minimal clinically important differences) in symptom severity (15-item Patient Health Questionnaire)^36^ and physical functioning (RAND 36-item Short Form Health Survey — physical component summary)^37^ were approached.

In total, 21 patients were approached by phone by two of the authors. Two patients were not willing to participate because of personal reasons; three patients declined participation because of time constraints. One patient cancelled the interview appointment for work-related reasons. Fifteen patients agreed to participate and were interviewed. All selected patients provided written informed consent.

All recruited patients experienced (episodes of) severe PSS, and most experienced symptoms for an extensive period of time (>5 years). The nature of symptoms varied. Almost all (*n* = 14) had symptoms in at least two symptom clusters, and a substantial number (*n* = 10) in at least three symptom clusters. Details about the symptoms experienced and other characteristics of the patients are shown in Table 1.

**Table 1.** Patient characteristics

**Variable**	**Values**
**Age, years, mean (range)**	55.4 (32–73)

**Sex, *n*/*N***	
Male	3/15
Female	12/15

**Education, *n*/*N***	
Higher educational level	4/15
Intermediate educational level	4/15
Lower educational level	7/15

**Location, *n*/*N***	
Rural	5/15
City	10/15

**Recruitment setting, *n*/*N***	
General practice	12/15
Specialised PSS programme	3/15

**Symptoms, *n*/*N***	
Fatigue	12/15
Musculoskeletal pain	12/15
Headache	6/15
Gastrointestinal symptoms (for example, nausea, abdominal discomfort)	5/15
Cardiopulmonary symptoms (for example, palpitations, atypical chest pain)	3/15
Dizziness	3/15

**Fluctuations/stability, *n*/*N***	
Fluctuations in symptom severity and physical functioning	9/15
Stable in symptom severity and physical functioning	5/15
Fluctuations in symptom severity, stable in physical functioning	1/15

*PSS = persistent somatic symptoms.*

### Data collection

The interviews used in the current study took place between January and April 2019. Based on the preference of the patient, 11 interviews were conducted at the patients’ home and four at the research department of the university in a private meeting room. All interviews were digitally recorded. Interviews lasted 60 min on average (range: 33–93 min). Patients received a €15 gift voucher.

Participants were informed that the main interviewer was a GP registrar and researcher, and the second interviewer was a medical intern involved in a research project on PSS. Both interviewers were female. The first received training in qualitative research and was supervised by an experienced qualitative researcher. Interviews were loosely structured using a topic guide with relevant areas that were explored in depth.

Open-ended questions were employed and patients encouraged to talk freely about their experiences and expand on any aspects they felt were relevant. The initial topic guide consisted of five main topics:

- the course of symptoms patients had experienced and how these interfered with their daily activities, with a special focus on stability and fluctuations over time (days, weeks, months, year(s));
- the factors contributing to fluctuations in symptoms;
- the management of symptoms and fluctuations;
- the role of patients’ social and work environment; and
- the role of the healthcare system and care providers.

After analysing the first two interviews, it was discovered that the (lack of) coordination of care was an important topic in patients’ narratives, and the following topics were therefore added:

- the need for/experience of coordination of care by healthcare professionals (HCPs); and
- their experience of and their preferred role for the GP in the organisation and delivery of care.

All participants received a summary of the interviews afterwards and were in agreement; thus no major changes in content were made.

### Data analysis

All interviews were transcribed verbatim and coded using Atlas.ti version 7. As indicated previously, this study is a secondary analysis of interviews collected in 2019: the first aim was to explore patients’ experiences of fluctuations in their PSS over time.^35^ For this analysis, the focus is on the parts of the interviews in which patients spoke about their experiences of the healthcare system and care providers, and more specifically the need for/experienced coordination of care from HCPs, and the preferred role of the GP in the coordination of care.

Data saturation on this matter was not aimed for, but instead the aim was to identify important key themes. In this study the analysis was based on thematic analysis as described by Braun and Clarke.^38^ As fragmented care played an important role in the illness narratives of the first two participants who were interviewed, this may have fuelled a preconception on the part of the researchers. To limit the impact of this preconception, open-ended questions were used and the researchers were conscious of this preconception when analysing the data. At least two authors (of the first three authors) were involved in all phases to enrich the analysis. All results were discussed in the research team. Finally, the report was produced, and quotes related to the themes were extracted.

## RESULTS

**Figure 1.**
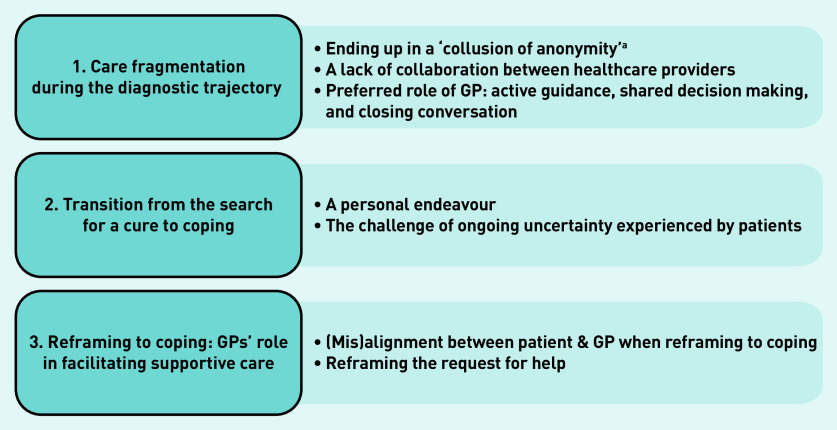
*Overview of themes.* *^a^A cycle of cross-referrals between specialists as first described by Michael Balint.^33^*

Three overarching themes in the illness trajectories important in the organisation and delivery of care were identified (Figure 1).

- care fragmentation during the diagnostic trajectory;
- transition from the search for a cure to coping; and
- reframing to coping: GPs’ role in facilitating supportive care.

Sub-themes related to these overarching themes are discussed here.

### Care fragmentation during the diagnostic trajectory

In line with the restitution narrative, the search for a diagnosis or explanation and cure for a patient’s symptoms was a starting point in all illness trajectories. Although it varied, most participants experienced a rather extensive and lengthy diagnostic trajectory for their PSS, resulting in a series of aborted trajectories. Within this search, patients in this study faced challenges concerning organisational factors, such as when seeing several GPs in a merged or larger practice, guidance, and communication between HCPs.

#### Ending up in a ‘collusion of anonymity’

Some patients in this study felt that no healthcare professional took the lead during the diagnostic trajectory and they ended up in a ‘collusion of anonymity’, being stuck in this cycle of cross-referrals from one specialist to another:

*‘Nobody knows. And they refer you to the next one. And that went on for such a long time* […] *I really thought “just talk to each other”, you know. Explain “this is a patient, I’ve ruled out this and this”. Because you’d be referred to the next one and he’d have the nerve to start talking about what the other one just ruled out.’*

(Participant [P]3, female, 30–35 years, recruited from specialised [S] PSS programme, PSS duration >5 years)

Participants assumed that the GP would receive information from the medical specialists and be updated on the diagnostic developments — although some experienced that this was in fact not the case:

*‘You don’t even think of going back to that GP, because you have now started the trajectory he* [GP] *put you on* […] *They’ll say “we will send a letter to your GP”. As a patient you think, at least I did, “well, that’s good, then he is informed”* […] *But then when I went there* [to the GP] *the next time, for something completely different, he said “Oh, you’ve seen a lot of specialists lately.” “Yes, that’s right, you referred me.” “Oh yes, now that you mention it”* […] *I really think the GP should play an active role here.’*

(P3, female, 30-35 years, recruited from S-PSS programme, PSS duration >5 years)

#### Risk of misalignment when seeing several GPs

Participants who were confronted with several GPs during their diagnostic trajectory experienced this as hindering, because they felt that nobody had a clear overview and, subsequently, misalignment in the approach to take could arise:

*‘I am in this duo practice, with two GPs who run the practice. What happens is that you go back and forth between them* […] *Of course they keep their notes in the computer, but they read a different story in these notes. And this can result in … how would you call that … differences.’*

(P8, male, 60–65 years, recruited from general practice [GP], PSS duration >5 years)

#### Contradictory information

In their search for a diagnosis, patients in this study regularly had to deal with contradictory information from the HCPs they encountered. In these situations, physicians and other HCPs disagreed on the diagnosis for the PSS, leaving some patients puzzled:

*‘So, I don’t have arthrosis at all* […] *“No,” he said, “it’s fibromyalgia.”* […] *So, then you don’t have what you thought you had, but I had to process that, the fact that “I don’t have arthrosis”.’*

(P12, female, 70–75 years, recruited from GP, PSS duration >5 years)

#### Guidance in the diagnostic trajectory: role of the GP

Patients in this study appreciated it when the GP takes an active role during the diagnostic trajectory. They indicated that the GP was the preferred person to take the lead in coordinating the diagnostic trajectory, as all information eventually ends up with the GP. Some patients had a GP who actively asked the patient to come back after the consultation with the medical specialist. For some patients, this was guided by a clear plan drawn up together with the GP before the referral. This active role of the GP and the shared decision making were experienced as positive, making the patient feel they are being taken seriously, while also maintaining a certain degree of control:

*‘In the last six months I feel I am being taken very seriously* […] *Now we have agreed to wait and see for a while, in particular regarding the pain in my chest; so, when I do come in with symptoms now, no nonsense, straight to the cardiologist* […] *Well, she agreed with that, so that’s what we do now.’*

(P8, male, 60–65 years, recruited from GP, PSS duration >5 years)

One patient indicated she would have appreciated a ‘closing conversation’ at the end of the diagnostic trajectory with her GP, to reflect on her search for a diagnosis and cure, and help her find closure with the fact that there was no readily available cure for her illness:

*‘I had expected that she* [GP] *would be the linking pin and all information would come back to her* […] *At some point I decided to go back to her to tell her “well, the conclusion was that there’s nothing they can do”. And I sort of expected that she would take the initiative for a kind of final, concluding conversation. But that doesn’t happen either* […] *Nobody really takes the lead.’*

(P4, female, 45–50 years, recruited from GP, PSS duration >5 years)

#### Communication between HCPs: preventing a ‘collusion of anonymity’

Some participants mentioned that communication between HCPs may play a crucial role in breaking the cycle of aborted diagnostic trajectories and shifting towards a more holistic approach to their PSS:

*‘They all just look at their own thing. But I actually don’t fit into anyone’s thing* […] *And if they had just discussed that beforehand or at some point along the way, I think the circle would have been closed sooner.’*

(P3, female, 30–35 years, recruited from S-PSS programme, PSS duration >5 years)

### Transition from the search for a cure to coping

Although some of the patients interviewed continued their search and remained dedicated to finding a diagnosis and cure, others acknowledged the unexplained nature of their symptoms, ended their search, and shifted focus towards coping with their PSS the best they could.

Moving away from the restitution narrative towards focusing on symptom management in itself was described as challenging.

#### The transition to coping (‘flipping the switch’): a personal endeavour

Several patients literally described the transition from searching for a diagnosis or explanation and cure for their symptoms towards coping the best they could as *‘flipping the switch’*, and underlined that this was a ‘personal endeavour’:

*‘So, it’s just a switch you have to flip. And if you don’t, you’ll never ever find a way out. Because you have to do it yourself.’*

(P1, female, 50–55 years, recruited from S-PSS programme, PSS duration >5 years)

#### The transition to coping: the challenge of ongoing uncertainty

Patients spoke about their ambivalent feelings regarding the uncertainty they had to deal with when making the transition: the small possibility that a rare diagnosis had been missed, or that new findings and scientific developments would eventually result in a diagnosis, stayed in the back of their mind:

*‘Yes, they said “we cannot find anything”. Then you have to believe that. You have to believe just that. They say that I am physically healthy. I take that for ninety per cent. And for ten per cent it remains open … and … well … no one can take that away from me. So … I leave it there. Within health care there may be a part that we just haven’t figured out. The knowledge is just not there yet.’*

(P1, female, 50–55 years, recruited from S-PSS programme, PSS duration >5 years)

### Reframing to coping: GPs’ role in facilitating supportive care

When patients visited their GP for coping strategies and supportive care, the extent to which patients were aligned with their GP in their focus on coping strategies and supportive care seemed to play a role in whether a patient’s request for supportive care was met.

#### Misalignment when reframing to coping: hindering supportive care

Some patients in this study experienced that their GP downplayed their symptoms when there was no diagnosis, obstructing further supportive care and symptoms management plans:

*‘I have had a GP and other people saying “but then there’s nothing wrong with you”. But you walk in my shoes for a day, it’s not nothing* […] *I have also heard things like “just keep going” or “put your back into it and you will be better in six months”. And that made me think “well, that’s what I’ve been trying for a couple of years now”.’*

(P3, female, age range 30–35 years, recruited from S-PSS programme, PSS duration >5 years)

#### Alignment when reframing to coping: ‘working it out together’

When the patient and GP seemed to be aligned in their focus on coping, patients reported positive experiences of searching for possible solutions and ways to cope with their symptoms together with their GP:

*‘It’s searching for things that may or may not help, together with my GP. It feels good that we decide together. That’s really important. At least you’re being heard.’*

(P11, female, 50–55 years, recruited from GP, PSS duration >5 years)

These patients underlined the importance of having a good relationship with their GP, in which they feel understood and supported, in order to turn to them for help and advice regarding symptom management. One patient described the experience with her previous GP as opposed to her current GP. Her previous GP did not seem to understand why she had consulted him for her PSS. With her current GP; however, she felt aligned and able to engage in discussing supportive care, something she appreciated:

*‘With my old GP I felt like he was thinking “I don’t know what to make of it, so what are you doing here?” And she* [new GP] *is someone who listens and helps to think along with me.’*

(P4, female, 45–50 years, recruited from GP, PSS duration >5 years)

#### Reframing the request for help: from curing to coping

Reframing the request for help was mentioned as a strategy to reach alignment with their GP when reframing and coping. A patient described how he rephrased the request for help as he moved away from the restitution narrative and was searching for strategies to cope with his PSS:

*‘If nobody brings up a solution, you continue your search. Then you will end up with a different story at the doctor. Not like “So I have a back pain, please do a check-up.” But you come to the doctor with the story “I have a back pain again, this didn’t help, I tried this, so what do we try next? You tell me.” Rephrasing those requests for help. Making your question clearer.’*

(P13, male, 40–45 years, recruited from GP, PSS duration >5 years)

## DISCUSSION

### Summary

This qualitative study aimed to explore the experiences of patients with PSS regarding coordination of care over the course of their illness trajectory. Three overarching themes were identified: care fragmentation during the diagnostic trajectory; transition from the search for a cure to coping; and reframing to coping: GPs’ role in facilitating supportive care.

Patients with PSS in this study described fragmentation in care, received contradictory information during the diagnostic trajectory, and underlined the importance of communication and collaboration among HCPs. Proactive guidance by their GP and shared decision making were positively valued. Switching the focus from finding a diagnosis and cure towards coping with symptoms was described as a ‘personal endeavour’ and made more challenging by the ongoing uncertainty. When patients made the transition, the extent to which they were aligned with their GP in their focus on coping seemed to play a role in whether their request for supportive care was met.

### Strengths and limitations

To the authors’ knowledge, this is the first qualitative study focusing on the experiences of patients with PSS regarding coordination of care and the GP’s role in this coordination. A strength of this study is the fact that patients were recruited throughout the Netherlands and varied in their demographic and social characteristics, and the course and diversity of their symptoms. An important limitation is the fact that this was a secondary analysis of qualitative interviews. The interviews were not primarily focused on the topic of coordination of care so the study did not aim for data saturation on this matter, but instead at identifying important key themes. Another limitation is the possible selection bias in the patients agreeing to take part in this study: most had experienced PSS for many years.

### Comparison with existing literature

Kornelsen *et al* have described how many patients with PSS get lost in the medical system and experience miscommunication between HCPs as *‘referrals got lost, consultation reports never returned to the initiating physician, and test results remained uncommunicated’*.^39^

Patients with PSS currently remain at risk of ending up in a cycle of specialised referrals described by Balint as a ‘collusion of anonymity’.^33^ Although aborted diagnostic trajectories are to some extent inherent to the illness trajectories in patients with PSS, the need for guidance, communication, and collaboration between HCPs described in the current study is in line with these prior findings. Although participants in the current study indicated that the GP was the preferred person to take the lead, the problem with the ‘collusion of anonymity’ is that the GP is often sidelined by the HCPs in secondary care. GPs and other HCPs should be aware of these issues and the importance of collaboration when referring patients to other HCPs.

The patients with PSS in the study by Kornelsen *et al*, like the patients in this study, started with an active search for a diagnosis and gradually moved towards acceptance of uncertainty. The therapeutic relationship seems important when dealing with this uncertainty.^39^ Nettleton *et al* showed that the narratives of patients with PSS were ‘chaotic’ and characterised by confusion and uncertainty, as there was no proper restitution narrative available. Patients in their study acknowledged that diagnosis is difficult and an explanation not always possible, but they were more concerned about securing some form of ongoing medical and social support.^27^

The patients with PSS in the current study faced challenges in gaining access to supportive care. Not only did the patient need to make the transition to coping, but their GP also had to be sensitive enough to respond to their request for help, and able and willing to offer coping strategies and supportive care. Stone^40^ has described that GPs, like patients, face difficulties reframing the ‘chaos’ and facilitating the transition to coping with a poorly defined illness. GPs needed to also tolerate uncertainty and faced challenges in ‘shifting gear’ from curing disease to coping with illness. Accepting responsibility for care and unconditional positive regard were among the strategies GPs used to manage the care of patients with PSS. Some GPs in the study by Stone described a deliberate shift in focus to care coordination, as they gave up their role of ‘technical expert’.^40^ The findings in the current study underline the need for a deliberate shift to supportive care by patients in alliance with their GPs. This will subsequently facilitate the change in focus and meet the supportive care needs of patients severely affected by their PSS.

### Implications for research and practice

In future research, it could be useful to study care usage of patients with PSS more extensively (both within and outside of the medical system) and how collaboration between different involved HCPs takes place. In addition, it would be interesting to examine strategies about how patients and GPs can ‘reframe to coping’ together and how GPs (or other central HCPs) can support patients with PSS to make the transition from the search for a cure to coping.

Future studies could also address the obstacles and barriers in care coordination and collaboration for patients with PSS, both from the HCPs’ and the patients’ viewpoints (including, for example, funding and organisational aspects of care).

In healthcare systems where the GP acts as a gatekeeper, it is recommended that GPs provide proactive guidance in the diagnostic process and that medical specialists and other HCPs strive for adequate communication and collaboration to prevent a ‘collusion of anonymity’. Moving away from the restitution narrative in a culture that prefers these narratives can be unsettling for patients and GPs alike, especially in the light of ongoing uncertainty.

The authors of the current study recommend that GPs are sensitive and supportive to patients who make the transition to coping with their symptoms by providing them with supportive care in a process of shared decision making. This may eventually improve long-term care for patients with PSS.
